# A fresh look at the Hyolithid *Doliutheca* from the Early Cambrian (Stage 4) Shipai Formation of the Three Gorges Area, Hubei, South China

**DOI:** 10.3390/biology11060875

**Published:** 2022-06-07

**Authors:** Fan Liu, Christian B. Skovsted, Timothy P. Topper, Zhifei Zhang

**Affiliations:** 1 State Key Laboratory of Continental Dynamics, Shaanxi Key Laboratory of Early Life and Environments, Department of Geology, Northwest University, Xi’an 710069, China; liufan@nwu.edu.cn (F.L.); christian.skovsted@nrm.se (C.B.S.); or timothy.topper@nwu.edu.cn (T.P.T.); 2 Department of Palaeobiology, Swedish Museum of Natural History, Box 50007, SE-104 05 Stockholm, Sweden

**Keywords:** hyoliths, Cambrian, South China, taxonomy, taphonomy

## Abstract

**Simple Summary:**

*Doliutheca orientalis* is revised from the Shipai Formation (early Cambrian Stage 4) of the Three Gorges area, South China. Specimens are preserved as casts in silty mudstone and as small shelly fossils in calcareous pelites. SEM and Micro-CT analyses show that *Doliutheca* possessed hyolithid-like skeletons (especially regarding the operculum) but significantly lack helens. This combination of features sees the genus placed within the Family Paramicrocornidae, a group of hyoliths closely related to hyolithids. Newly compressed specimens of *Doliutheca* from silty mudstone show some soft-part preservation with the gut clearly visible. Additionally, the highly variable apex morphology of *Doliutheca* is explained as a consequence of differences in preservation due to secondary deposits formed inside the shell. This finding highlights the challenge of preservational artefacts in calculating the disparity and diversity of early Cambrian skeletal fossils, and the resulting potential effects on taxonomic assessments on the diversity of skeletal taxa in the Cambrian.

**Abstract:**

New hyolith specimens from the early Cambrian (Stage 4) of the Three Gorges area, western Hubei Province are described and assigned to the species *Doliutheca orientalis*. *Doliutheca* are preserved in two taphonomic modes: casts in silty mudstone revealing gross morphology and some soft parts, and internal molds in calcareous pelites, which exhibit new morphological details of the conch and operculum. SEM and Micro-CT analyses show that *Doliutheca* preserve well-developed platy clavicles and cardinal processes on the interior of the operculum composed of rod-shaped tubular elements. This observation and the distinct cardinal and conical shields of the operculum indicate that *Doliutheca* could be placed within the Family Paramicrocornidae, most recently established as a group of hyoliths closely related to hyolithids.

## 1. Introduction

Hyoliths were diverse and abundant benthic marine lophotrochozoan animals in the Paleozoic, ranging from the early Cambrian to the late Permian [1,2,3]. Hyoliths have conical aragonitic shells and are generally separated in two groups: Orthothecida and Hyolithida [1,3,4]. Typical hyolithids have three skeletal parts including a conch, an external operculum and two lateral spines called helens [1,5,6,7,8,9]. Orthothecids, on the other hand, consist of a conch and a retractable operculum without helens [1,5,6,9]. However, some Cambrian hyoliths have proved difficult to easily place in either of these groupings [10]. The family Paramicrocornidae was recently erected to accommodate taxa that display a hyolithid-like morphology but lack helens [11,12,13]. Liu et al. [13] suggested that Paramicrocornidae are the closest relatives of the Hyolithida, representing an intermediate stage of hyolith evolution [11,12,13].

*Doliutheca* Qian, 1989 from South China, is one such cryptic hyolith with an uncertain higher order affinity that is commonly preserved in small shelly fossil (SSF) assemblages. The taxonomic history of the genus and related species has been complicated [14,15,16] and *Doliutheca* is in need of revision. Here, we present new hyolith material at the Xiachazhuang section of the Three Gorges area, Hubei Province [17], which yields abundant fossils from the early Cambrian (Stage 4) Shipai Formation. The hyoliths, here identified as *Doliutheca orientalis* (Qian, 1978), are noteworthy as the specimens which are preserved in two taphonomic states: compressed specimens preserved on bedding planes in silty mudstone and internal molds preserved as small shelly fossils (SSF) in acid disaggregated calcareous pelite layers. Specimens on bedding planes preserve the general conch morphology as well as limited soft tissues (especially the gut), and the SSF specimens preserve delicate internal structures of both conch and opercula.

## 2. Geological Setting and Stratigraphy

**Figure 1:**
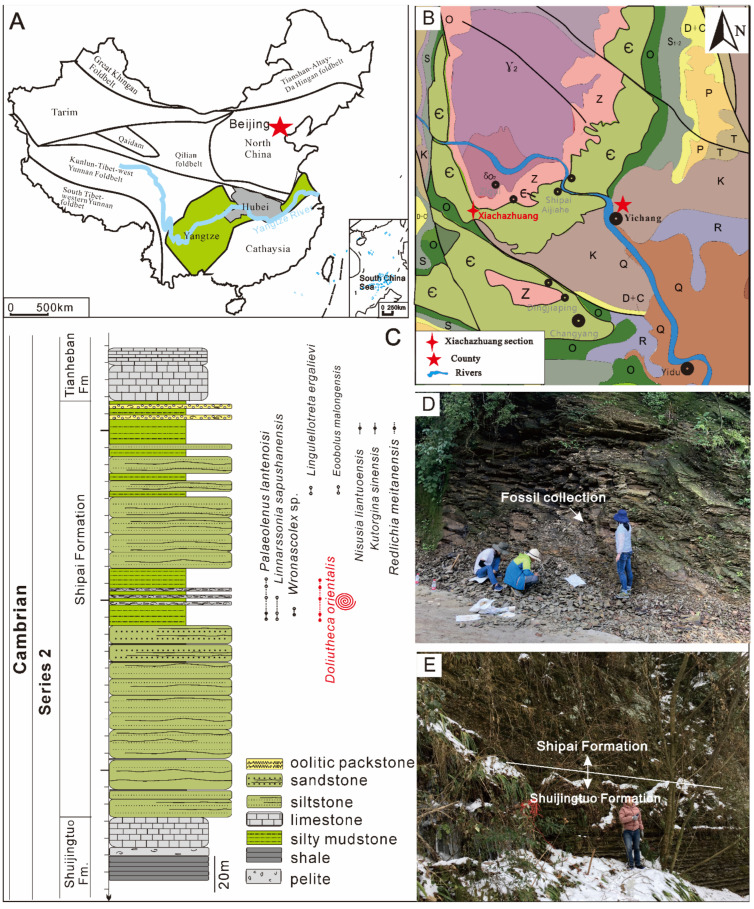
Simplified geological map, paleogeography, fossil localities and early Cambrian stratigraphy of the Three Gorges area. (**A**) Generalized map showing the principal continental blocks of China. (**B**) Geological map notes the localities of the Xiachazhuang section in the Yangtze Gorges region. (**C**) Stratigraphic column with the early records of fossil biozones from the Shipai Formation of the Xiachazhuang section, noting the levels where hyoliths illustrated in this paper were recovered (marked by the arrow). (**D**) Field photo of the Shipai Formation of the Xiachazhuang section, which yielded abundant hyoliths and other fossils including brachiopods, trilobite, etc. (**E**) Field photo showing the boundary between the Shuijingtuo Formation and the upper Shipai Formation.

Cambrian strata are widely distributed on the Yangtze Platform (Figure 1A), situated between the Qinling orogenic belt to the North and the Cathaysia suture to the Southeast. Previous studies on the richly fossiliferous Shipai Formation in the Three Gorges area of Hubei Province in the northern part of the Yangtze Platform have focused on chemostratigraphy [18] and documented shelly fossils and abundant soft-bodied fossils including *Vetulicola*, brachiopods and palaeoscolecids [17,19,20,21]. Based on evidence from brachiopods [20,21] and trilobites (corresponding to the *Redlichia meitanensis* and *Palaeolenus lantenoisi* zones), the Shipai Formation was correlated with the Canglangpuian Stage, that equates with Cambrian Stage 4 [19,20,22].

The Shipai formation (Figure 1C) is mainly composed of yellowish-green silty mudstone and thin bedded limestone containing abundant fossils, including *Redlichia meitanensis*, *Palaeolenus lantenoisi* and brachiopods [23]. The material described here is derived from the Xiachazhuang Section, Maoping town, southwest Hubei Province, where the Shipai Formation is richly fossiliferous [17,21].

**Figure 2:**
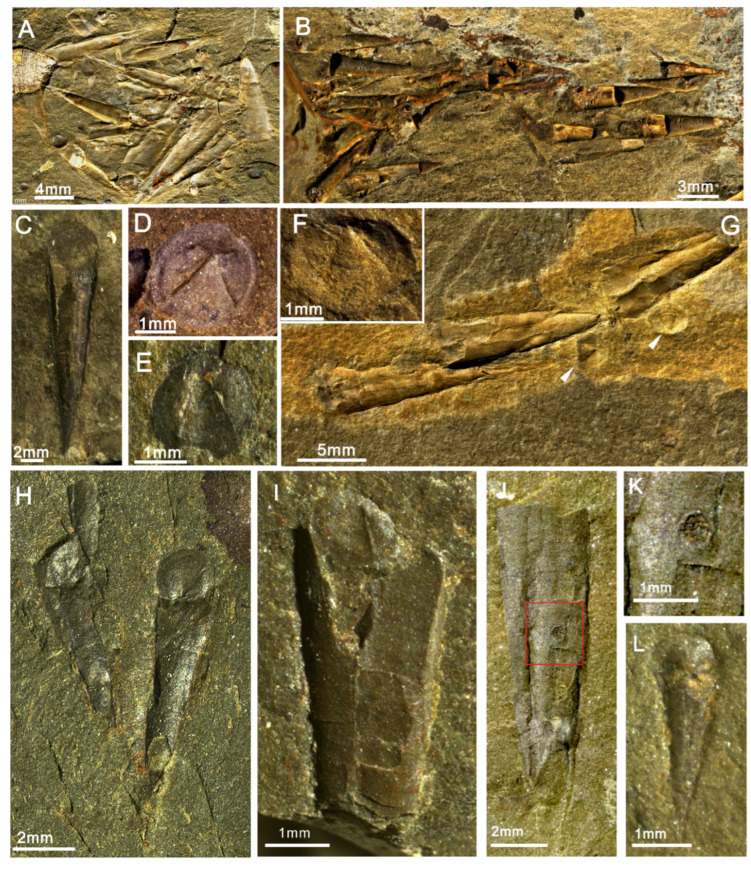
Hyolith *Doliutheca orientalis* from the Shipai Formation of the Xiachazhuang section, Hubei Province, China. (**A**,**B**) Concentrations of *Doliutheca orientalis*. (**A**) ELI QJP-SP-H-125. (**B**) ELI QJP-SP-H-167. (**C**) The ventral view of *Doliutheca orientalis* showing the arch-shaped ligula. ELI QJP-SP-H-293. (**D**–**F**) the rounded opercula of *Doliutheca orientalis*, showing the X-shaped imprint of strongly developed clavicles and cardinal process. (**D**) ELI QJP-SP-H-128. (**E**) ELI QJP-SP-H-343. (**F**) ELI QJP-SP-H-067. (**G**) Three individual conchs of *Doliutheca orientalis* with associated opercula. ELI QJP-SP-H-067. (**H**–**I**) *Doliutheca orientalis* with articulated operculum. (**H**) ELI QJP-SP-H-368. (**I**) ELI QJP-SP-H-382. (**J**) Hyolith conch with possible predatorial borehole or the trace from an epizoan/epicole. ELI QJP-SP-H-009. (**K**) The close-up view of hole in (**J**). (**L**) The dorsal view of hyolith operculum showing distinct cardinal shield and conical shield. ELI QJP-SP-H-325.

Three stratigraphic horizons of the Shipai Formation yielded fossils at the Xiachazhuang section (Figure 1C); (1) a green-yellowish silty mudstone situated ca. 120 m above the base of the section, that belongs to the *Palaeolenus lantenoisi* trilobite Zone (Figure 1D); (2) a green-grayish siltstone, with abundant specimens of the brachiopods *Eoobolus* and *Lingulellotreta* ca. 30 m above horizon 1; (3) the top of the Shipai Formation, that yields brachiopods such as *Kutorgina* and *Nisusia* ca. 50 m above horizon 2. The majority of hyolith specimens were collected from horizon 1. This level yields a rich fossil assemblage including brachiopods [21], hyoliths, arthropods, palaeoscolecids, sponge spicules and chancelloriid sclerites [17]. Three calcareous mudstone layers (each layer approximately 10 cm thick), that preserve a variety of SSFs including hyoliths, brachiopods, molluscs, bradoriids and trilobites, were also found and interspersed in the middle of the green-grayish siltstone (Figure 1C).

## 3. Materials and Methods

In total, 381 compressed fossils on bedding planes were investigated. Selected specimens were examined under a binocular Zeiss Zoom Stereomicroscope (Carl Zeiss Microscopy GmbH, Germany) and photographed with a stereophotographic Zeiss Smart Zoom 5. Petrographic thin sections were made to examine skeletal fossils under a polarized microscope, and these were photographed using a stereomicrograph system Nikon SMZ1500 at the Department of Geology, Northwest University. Selected hyolith specimens from mudstone were analyzed through μ-XRF (Bruker M4 TORNADO non-destructive technique Micro X-ray Fluorescence) (Billerica, MA, USA) to determine elemental composition.

Thousands of SSFs were recovered through acetic acid (~10%) dissolution of samples from pelitic layers collected from the middle Shipai Formation. Specimens were handpicked from the undissolved rock residues and selected specimens were gold coated and studied further using scanning electron microscope (SEM FEI Quanta FEG450) (FEI, Hillsboro, OR, USA) facilities at Northwest University (FEI, Hillsboro, OR, USA). One specimen was examined using the micro-CT scanner (ZEISS Xradia 520 Versa) (Carl Zeiss, Meditec, Inc., Dublin, OH, USA) at Northwest University. The three-dimensional reconstructions of the specimen were based on nearly 1000 single images equally spaced through 360° by using the computer software V.G. Studio 3.2 Max.

## 4. Systematic Paleontology

The specimens discussed and described here, including the compressed fossils from the silty mudstone and SSFs from pelitic layers, are deposited in Department of Geology, Northwest University.

Class Hyolitha Marek, 1963
Family Paramicrocornidae Liu, Skovsted, Topper & Zhang, 2022

**Discussion.** Paramicrocornidae was proposed as a unique group of hyoliths, possessing a hyolithid-like morphology but notably lacking helens [13]. This family is characterized by a straight, conical-pyramidal conch, aperture with a short ligula but without lateral sinuses as well as a triangular to oval operculum with distinct cardinal and conical shields and strongly developed cardinal processes and clavicles composed of a palisade-like arrangement of parallel rods. As defined by Liu et al. [13], Paramicrocornidae includes two genera, *Paramicrocornus* Qian, Xie and He, 2001 [16] from the Shuijingtuo Formation of South China, and *Protomicrocornus* Pan, Skovsted, Sun and Li, 2019 [24] from Houjiashan Formation of North China.

The rod-like structure of the platy clavicles in Paramicrocornidae was suggested to be a precursor to the characteristic helens that define hyolithids, forming an evolutionary link between the orthothecids and the hyolithids [12]. A recent phylogenetic analysis of Cambrian and Ordovician hyoliths confirms this evolutionary pattern with a monophyletic hyolithid group derived from a paraphyletic Orthothecida, with Paramicrocornidae as the closet relatives of typical hyolithids (bearing helens) [13].

**Figure 3:**
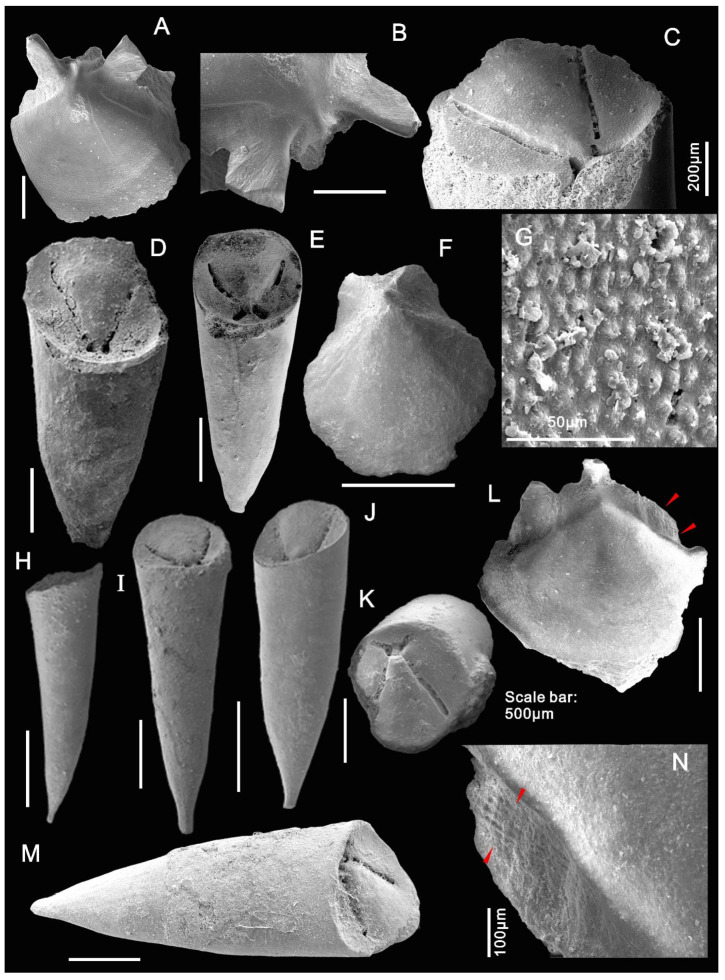
Internal moulds of *Doliutheca orientalis* from SSF assemblages of the Shipai Formation at the Xiachazhuang section, Hubei Province, China. (**A**,**B**) Mold of operculum in external view noting prominent cardinal process in the enlarged view in (**B**). ELI QJP-SP-H-SSF-8101001. (**C**) The granular texture on the operculum imprint. ELI QJP-SP-H-SSF-81019. (**D**–**E**) Internal molds with conch and opercula, showing the X-shaped imprint caused by the clavicles and cardinal process. (**D**) ELI QJP-SP-H-SSF-61035. (**E**) ELI QJP-SP-H-SSF-816001. (**F**) external view of operculum, showing the convex conical shield and flat cardinal shield. ELI QJP-SP-H-SSF-7109. (**G**) Close-up of tubercles on the conch surface. ELI QJP-SP-H-611002003. (**H**) lateral view of conch showing the rounded smooth lateral transition between dorsum and venter and slight dorsal curvature of conch. ELI QJP-SP-H-SSF-811107. (**I**) Conch with articulated operculum in dorsal view. ELI QJP-SP-H-SSF-810405. (**J**) Conch with articulated operculum in dorsal view, note gradual expansion of conch from the sharp apex to aperture. ELI QJP-SP-H-8104039. (**K**) Apertural view of conch with articulated operculum, noting the rounded conch cross-section with deep imprint of operculum. (**L**,**N**) Internal mold of operculum showing the exposed clavicle with rod-shaped elements of the clavicle (marked by red arrows), with the close-up view in (**N**). ELI QJP-SP-H-SSF-8102017. (**M**) Lateral view of conch with articulated operculum. ELI QJP-SP-H-SSF-81010.

The new material of *Doliutheca* documented here shows a distinct operculum with differentiated cardinal and conical shields and wide plate-like clavicles composed of parallel rods and a conch with a short ligula on the venter without lateral sinuses. This indicates that *Doliutheca* is hyolithid-like in morphology but lacks helens (Figure 2 and Figure 3). Therefore, *Doliutheca* is assigned here to the recently proposed Family Paramicrocornidae Liu, Skovsted, Topper and Zhang, 2022.

Genus *Doliutheca* Qian, 1989

**Type species:***Doliutheca orientalis* (Qian, 1978), Cambrian Series 2, Hubei and Yunnan provinces, South China. The originally proposed type species [15]; *Paragloborilus capitalis* Jiang, 1982 [25], from the Zhongyicun member of Huize County, Yunnan Province, South China, is here considered a junior synonyom of *Doliutheca orientalis*.

**Species included**: Type species.

**Emended Diagnosis.** Long straight orthoconic conch with rounded cross-section. Apex dorsally curved. Ligula short and nearly semicircular. Sharp straight apex, slowly and regularly expanding towards conch aperture. Venter flat, domed dorsum with poorly defined median carina. Round operculum with platy clavicles and strongly developed cardinal processes arranged in an X-shape. Clavicles composed of closely set parallel rod-like units. Distinct, flat narrow cardinal shield and slightly convex conical shield. Transverse growth-lines well preserved on both venter and dorsum of conch and concentric growth lines faintly developed on operculum.

**Remarks:** Qian [14] reported three species from China and assigned to the genus *Doliutus* Missarzhevsky, 1969: *Doliutus orientalis* Qian, 1978, *Doliutus zhenbaensis* Qian, 1978 and *Doliutus* *shipaiensis* Qian, 1978. However, these species all show obvious differences from original material of *Doliutus* Missarzhevsky, 1969 (figures from [26]) from Siberia, especially concerning the gradually expanding conch, the non-orthogonal shape of the aperture and the significantly smaller ligula. Eventually, species *Doliutus zhenbaensis* Qian, 1978 was later referred to the new genus *Inflatatheca* [16], with the large and wide conch showing the lentoid-subtriangular cross-section and strong blunt juvenile shell ([16], Figures II16–18). Qian [15] proposed the new genus *Doliutheca* Qian, 1989, assigned to the order Circothecida Sysoev, 1968, with *Paragloborilus capitatus* Jiang, 1982 as the type species (= *Doliutheca capitatus)*. Furthermore, Qian [15] revised the previously described Chinese species of *Doliutus* and synonymized *Doliutus orientalis* and *Doliutus* *shipaiensis* based on the apparent similarities in conch morphology and assigned this material to *Doliutheca orientalis* (Qian, 1978). In his discussion, Qian [15] emphasized the similar conch morphology between *D. capitalis* and *D. orientalis*, but noted a transverse furrow at the transition from juvenile to adult conch and a flatter ligula in the type species. However, as demonstrated here, the perceived change between the juvenile and adult shell was likely based on the morphology of internal molds and does not reflect original shell morphology. Hence, we consider *D. capitalis* (Jiang, 1982) as a junior synonym of *D. orientalis* (Qian, 1978).

Based on the new material of *Doliutheca* from the Xiachazhuang section, we suggest that *Doliutheca* is closely comparable to *Paramicrocornus* and *Protomicrocornus* and should be assigned to the Family Paramicrocornidae [13]. All three taxa show a hyolithid-like morphology with a ligula on the conch aperture and an operculum with distinct cardinal and concial shields and strongly developed clavicles and cardinal processes composed of parallel rods, while lacking any evidence of helens or helen-related features on either operculum (rooflets and a wide gap between clavicles and cardinal processes) or conch (lateral sinuses). However, *Doliutheca* is differentiated from *Paramicrocornus* and *Protomicrocornus* by the more orthoconic and longer conch with rounded cross-section, the circular shape of the operculum that also hosts prominent cardinal processes and clavicles in an X-shaped arrangement.

**Stratigraphic and geographic range.** Shuijingtuo to Shipai Formation, Hubei; Zhongyicun Member, Yunnan; Early Cambrian, Terreneuvian-Series 2, South China.

**Figure 4:**
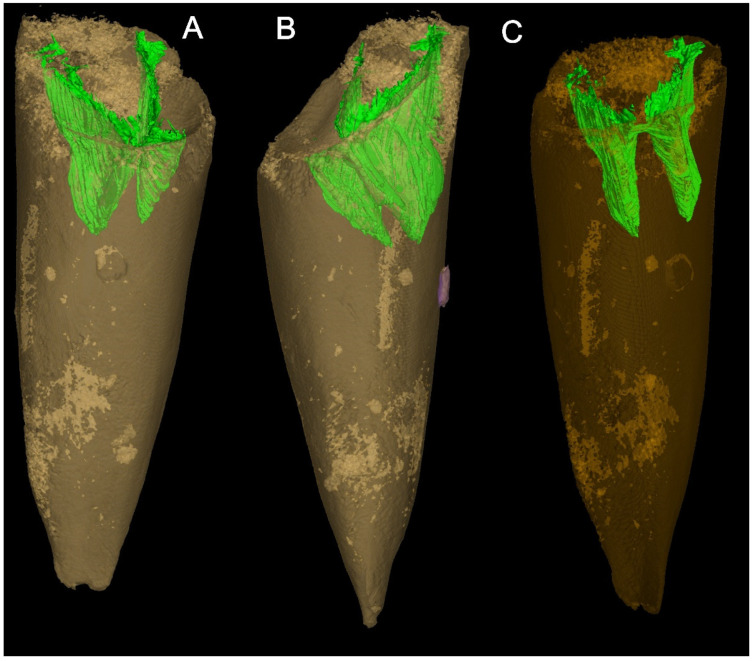
Reconstruction of *Doliutheca orientalis* from Micro-CT analysis (semi-transparent model), showing the 3D mold of platy clavicles (green) in the steinkern. (**A**,**C**), dorsal view; (**B**), lateral view. The purple part is the potential borehole.

*Doliutheca orientalis* (Qian, 1978)

(Figure 2, Figure 3 and Figure 4)

1978 *Doliutus orientalis* Qian, p. 27, 28, pl. 7, Figure 5 in [14].

1978 *Doliutus shipaiensis* Qian, p. 28, pl. 7, Figure 6 in [14].

1982 *Paragloborilus capitalis* Jiang, Luo et al., p. 167, pl. 13, Figures 21 and 22 in [25]

1989 *Doliutheca orientalis* Qian, p.76, PI. 5, Figure 12, 161.Pl. 7, Figures 1 and 2 in [15]

1989 *Doliutheca capitata* Qian, p.76, Pl. 7, Figure 3, 41 Pl. 79, Figures 7–91. Pl. 80, Figures 1–3 in [15]

2015 *Doliutheca capitata* Yang et al., p.1551. Figure 8P,Q in [27].

2020 *Doliutheca orientalis* Sun et al., p.4. Figure 2K in [28].

**Holotype:** Specimen 33736, a conch (Figure 7.5 in [14]) from the upper Shuijingtuo Formation, Hubei Province, South China.

**Material:** More than 800 specimens of smooth calcium phosphatic molds and casts from acid disintegrated pelite layers and 381 compressed hyolith specimens from silty mudstone of the middle Shipai Formation.

**Diagnosis.** Slender conch with short semi-rounded ligula on a slightly flat venter. Conch displays a rounded cross-section and the operculum is circular in outline. Operculum with distinct, narrow, well-developed cardinal and conical shields. Cardinal processes separated from the clavicles by a narrow gap and extend at right angles from the clavicles. The platy clavicles are composed of parallel rod-shaped tubular elements. Conch apex sharp and slightly curved towards dorsum. Faint growth lines preserved on the conch and concentric lines poorly visible on the operculum.

**Description:** Specimens from mudstone show the nearly complete morphology of the conch (Figure 2), sometimes with operculum articulated or at least in close proximity to the conch (Figure 2H,G). Straight conch with very thin, sharp apex, orthoconic to slightly cyrtoconic with apex curving toward dorsum, greatest preserved length 1.5 cm and width 2–3 mm. AD (angle of divergence) = 12–14° (Figure 2C–H). Apertural region is poorly preserved, although some specimens bear an arched ligula (Figure 2C). Sculpture on the external surface with faint transverse growth lines on dorsum (Figure 2A,J). Opercula are circular in shape, usually with the imprint of clavicles on the inner surface and faint concentric growth lines on the exterior surface (Figure 2E,F,H). Specimens in dorsal view show the distinct flat cardinal shield and convex conical shields separated by a narrow furrow (Figure 2L).

Small, slender and straight internal mold of conch from SSF assemblages is preserved with a short ventral ligula and the apical region slightly curved towards dorsum (Figure 3H,J). The specimens are about 5 mm in length (Figure 3D,E,H,I,J,M) with an apertural width and height of about 0.5 mm, AD (angle of divergence) average = 30.5° in apical region and AD average = 52° in non-apical region (N = 43 well-preserved specimens). The specimens are invariably phosphatic steinkerns and many are preserved with imprints of the inner surface of the operculum in the apertural region (Figure 3D,E,I,J,K,M). The dorsum is more inflated than the venter (Figure 3J,M and Figure 4). Dorsal–ventral transition smooth (Figure 3H,M).

The circular opercula show distinct cardinal and conical shields (Figure 2F,L and Figure 3A,F,L). The conical shield is longer and more convex than the cardinal shield (Figure 2L and Figure 3A,F,L). Molds of the internal surface of the operculum preserve imprints of narrow, blade-like clavicles and cardinal processes separated by a narrow gap (Figure 3C). Cardinal processes are aligned at roughly right angles to the cardinal processes giving a X-shaped impression (Figure 2D,E,I and Figure 3C,D,E,K). The platy clavicles are composed of numerous parallel rod-shaped tubular structures (Figure 3N). Micro-CT analysis of the casts of the operculum in internal molds shows deep impressions of the clavicles and cardinal processes (Figure 4). Faint concentric growth lines ornament the external surface of the operculum (Figure 2F). Internal surfaces are instead ornamented by small tubercular structures with average diameter of individual tubercles of approximately 4 µm (Figure 3C,E,G).

One hyolith conch in the investigated material preserves a possible circular borehole with countersunk margins (diameter: ca. 300 μm) situated close to the center of the dorsum at about mid length (Figure 2J–K). Similar beveled boreholes in Paleozoic fossils with countersunk edges have been suggested to be formed by predatorial attacks (radular boring) by mollusks, such as monoplacophorans and some gastropods [29,30,31]. However, due to the limited number of specimens available, no definitive conclusions can be reached regarding the origin of this circular hole.

**Remarks.** Qian, 1989 [15] included two species in *Doliutheca*, the species *Doliutheca orientalis* (Qian, 1978) and *Doliutheca capitalis* (Jiang, 1982). He remarked that the two species are similar in conch morphology, except for a transverse furrow that could be observed between the juvenile and adult shell of *Doliutheca capitalis*. However, this distinction is problematic as it was based on differences in the morphology of the internal molds of conchs and not the conchs themselves.

The new specimens of *Doliutheca* from SSF assemblages from the Xiachazhuang section preserve conchs with a variably shaped apex, but with uniformly rounded operculum with platy clavicles and cardinal process forming a X-shaped imprint (Figure 3). Combined with evidence from thin sections and specimens on bedding planes (see below Section 5.2), we conclude that morphological differences between the steinkerns of *Doliutheca orientalis* and *Doliutheca capitata*, as defined by Qian [15], were caused by the secondary thickening of the apical shell wall and does not reflect the original morphology of the conch interior (see below in discussion). The morphology of the apex is thus highly variable in *Doliutheca* internal molds, depending on the degree of preservation of the apical region. Thus, *Doliutheca orientalis* and *Doliutheca capitata* should belong to the same species. *Doliutheca orientalis* was the earliest reported species [14], so we regard *Doliutheca capitata* as a junior synonym of *Doliutheca* *orientalis*.

**Occurrence.** Shuijingtuo–Shipai formations, Cambrian Series 2, Hubei Province, South China; Zhongyicun Member of Yuhucun Formation, Terreneuvian of eastern Yunnan Province, South China.

## 5. Discussion

### 5.1. Hyolith Accumulations and Soft Part Preservation

**Figure 5:**
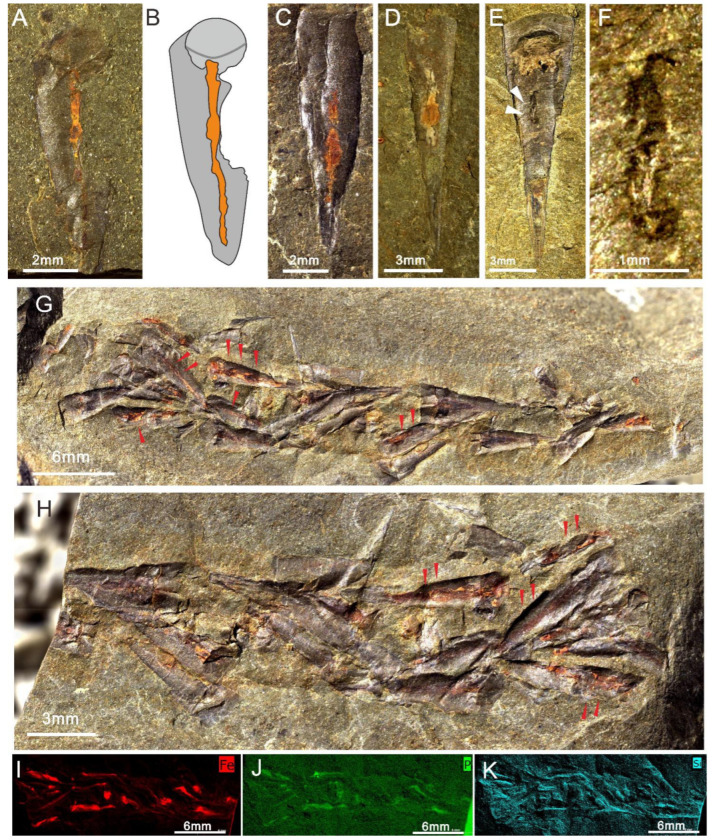
*Doliutheca orientalis* with soft-part preservation from the Shipai Formation of the Xiachazhuang section, Hubei Province, China. (**A**,**B**) One conch with articulated operculum with the digestive tract preserved as a red stain. ELI QJP-SP-H-379. (**B**) Interpretative sketch of (**A**), the digestive tract (orange) preserved in the conch (gray). (**C**,**D**) The gut of specimens preserved as a linear shape of reddish-brown color. (**C**) ELI QJP-SP-H-235. (**D**) ELI QJP-SP-H-343. (**E**,**F**) Hollow cavities composed of linear (marked by white arrows), dark-stained elements preserved in the conch center, ELI QJP-SP-H-029. (**F)** Close-up image of hollow cavities in (**E**). (**G**–**K**) Part and counterpart of hyolith concentrations, which preserved soft tissue in the disposition of the digestive system in the conchs (marked by red arrows). ELI QJP-SP-H-215a,b. (**I**–**K**) μ-XRF elemental mapping of iron, phosphorus and silicon, emphasizing the enrichment of iron.

Many specimens of *Doliutheca orientalis* from the silty mudstone of the Shipai Formation are preserved in dense aggregations, with orientations ranging from apparently random to conchs on single bedding planes showing some broad alignment in a single direction (Figure 2A,B and Figure 5G,H). In some cases, conchs articulated with the operculum are preserved together in ribbon-like accumulations, where multiple specimens preserve the reddish-brown traces of the gut tract (Figure 5A–H).

Shell accumulations are commonly preserved in Cambrian Lagerstätten [32,33,34]. Hyolith aggregates have been previously reported from the early Cambrian Chengjiang Biota, showing several different arrangements, with some of them considered as coprolitic in origin [35]. However, some, especially for unique pear-shaped aggregates, were suggested to have been a result of a detritus habit with hyoliths feeding on organic-rich materials or microbial films [36]. Herein, these articulated specimens of *Doliutheca orientalis* with soft tissue preserved indicate that this aggregation may have accumulated via water currents, rather than representing a coprolite. Abrasion on the surface of conchs in this assemblage suggests that hyolith specimens were transported slightly before a rapid burial.

The gut of *Doliutheca orientalis* is generally preserved as a straight, linear structure and is reddish in color and probably represents the rectum of the U-shaped hyolithid gut (Figure 5A–D). However, one specimen preserves a linear, black-stained cavity (Figure 5E,F), which is similar to preserved guts in hyolithids from the Guanshan Biota, where it was likely formed by the demineralization of soft parts that were originally calcified by microbial activity [37]. Elemental mapping of μ-XRF analyses indicates that conchs have been replaced by silica and phosphatic material, and that organic soft tissues are preserved predominantly as films of iron oxide showing the enrichment of iron (Figure 5I–K), presumably as a weathering product of pyrite [37].

### 5.2. Apical Variability

*Doliuthecaorientalis* from the Shipai Formation in the Xiachazhuang section exhibits two preservation states: compressed specimens hosted in the greenish silty mudstone and phosphatized, primarily internal molds in SSF assemblages from the calcareous pelite layers in the middle Shipai Formation, approximately one meter above the mudstone hosted material. The presence of the same hyolith taxon in two preservational states has the capacity to reveal new details that enhance our understanding of hyolith morphology and taxonomy.

**Figure 6:**
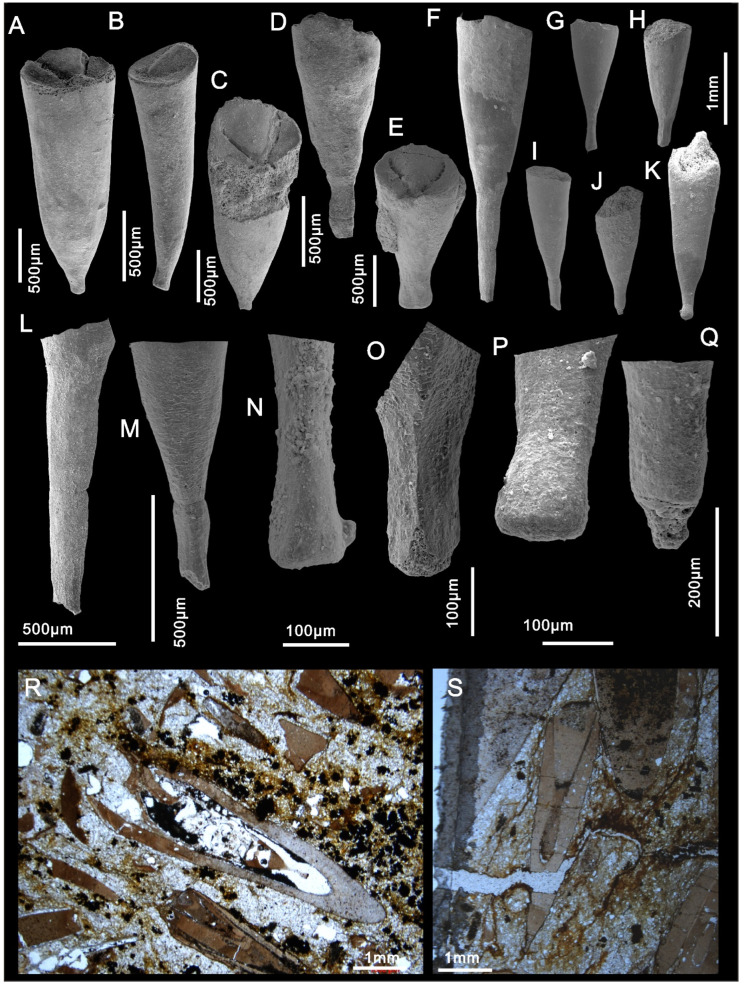
Variously shaped apical parts of *Doliutheca orientalis* conchs from SSF assemblages from the Shipai Formation of the Xiachazhuang section, Hubei Province, China. (**A**,**C**) Flat apical part of steinkern. (**B**) Disc-like-shaped apex. (**D**,**E**) Flat apex presumably resulting from the breakage of the distal part. (**F**–**H**) Sharp or tubular distal part. (**I**–**K**) Fusiform-shaped apex. (**L**) Close up view of (**F**). (**M**) The close-up view of (**I**). (**N**) The close-up view of (**G**). (**O**) The close-up view of (**H**). (**P**) The close-up view of (**K**). (**Q**) The close-up view of (**J**). (**R**,**S**) The hyoliths in the thin sections revealed the complete conch shell, the normal conical conch with the close apex, but the internal mold preserved a fusiform-shaped distal part. (**A**) ELI QJP-SP-H-SSF-81A2001. (**B**) ELI QJP-SP-H-SSF-81A4001. (**C**) ELI QJP-SP-H-SSF-81D7001. (**D**) ELI QJP-SP-H-SSF-81A16001. (**E**) ELI QJP-SP-H-SSF-0781039. (**F**) ELI QJP-SP-H-SSF-61016. (**G**) ELI QJP-SP-H-SSF-816027. (**H**) ELI QJP-SP-H-SSF-816045. (**I**) ELI QJP-SP-H-SSF-8110124. (**J**) ELI QJP-SP-H-SSF-811067. (**K**) ELI QJP-SP-H-SSF-61021.

Internal molds of *Doliutheca orientalis* conchs show a highly variable apical morphology and this has previously been used to inform taxonomic determination and the biological interpretation of hyoliths from the Shipai Formation [14,15]. In the majority of specimens, the apex is represented by a sharply pointed or tubular distal region (Figure 6A–Q and see Figure 5 in the [38]). However, in some cases the apex is fusiform with a slight distal expansion (Figure 6J,K,N,P). Other specimens preserve a flat, disc-like apex which is presumably a result from the breakage of a tubular or fusiform distal part (Figure 6A–E,L,M). The analysis of thin sections gives a more complete picture and reveals a simple conical conch outline combined with a thickened shell wall that surrounding a narrow tubular or fusiform apical canal (Figure 6R–S). A similar apex morphology is also visible in compressed specimens in mudstone (Figure 5D,E; See also discussion in Liu et al. [38]).

The apparent variability of the apex led Qian [15] to suggest that the different apexes correspond to two stages of ontogeny in *Doliutheca orientalis*, an elongated tubular larval shell and a bulkier adult shell. The new material described here shows that the variable shape of the “larval shells” reflect differential preservation rather than reflecting the original ontogeny of the taxon. The apical morphology of internal molds thus appears to be a consequence of secondary thickening of the apical shell wall and does not reflect the original morphology of the apical region. The presence of this secondary thickening in both mudstone and limestone hosted specimens suggests that it was biologically controlled. Many hyolith taxa are known to produce intermittent or regularly arranged internal septa [38,39,40] and the deposition of secondary shell material in *Doliutheca* may have served a similar purpose to seal off or strengthen the otherwise vulnerable apical region of the conch [6]. However, to investigate the original larval development of this taxon, it would be beneficial to examine the original shell (e.g., [41]) rather than the phosphatic internal molds.

## 6. Conclusions

**Figure 7:**
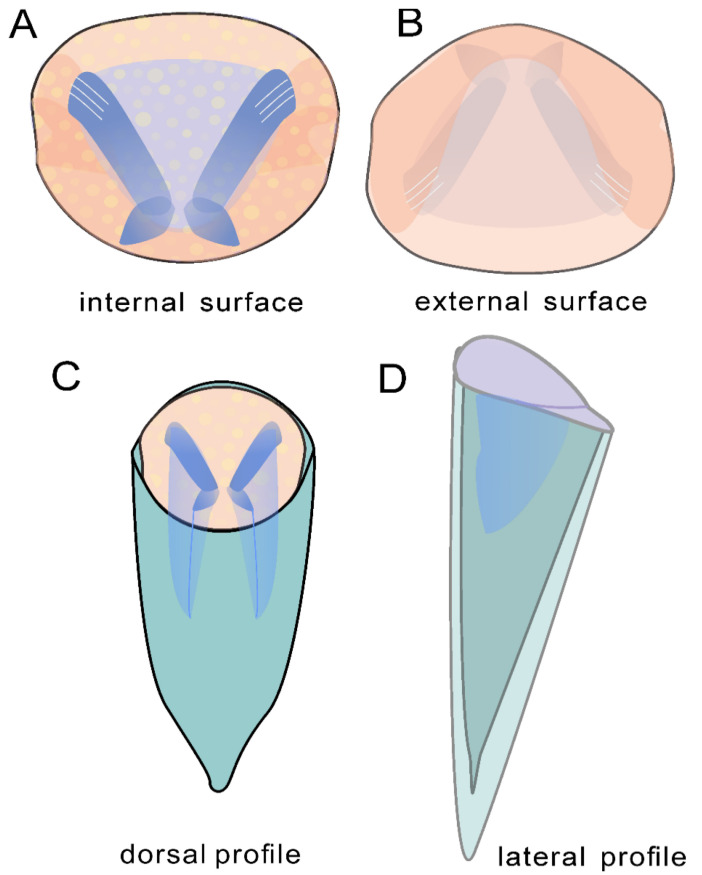
Simplified reconstruction of *Doliutheca orientalis* from the Shipai Formation of the Xiachazhuang section, Hubei Province, China. (**A**,**B**) Sketch of operculum. (**C**) The steinkern with articulated operculum, noting the platy clavicles. (**D**) Sketch of reconstruction showing *Doliutheca orientalis* with shell in lateral profile.

In this study, *Doliutheca* is revised (Figure 7) and assigned to the Family Paramicrocornidae based on the presence of a circular operculum with a narrow cardinal shield and platy clavicles composed of parallel rods, indicating that this genus was closely related to hyolithids, rather than orthothecids as suggested by Qian [15]. Our study of *Doliutheca* preserved in two taphonomic modes reveals new anatomical details and shows that the apex morphology of steinkerns, previously used to inform taxonomic assessment, is a consequence of differences in preservation due to secondary deposits formed inside the shell. It should be noted that future taxonomic work on hyoliths should exclude the effect of such preservational artefacts. This observation highlights the challenge of reducing the effect of taphonomic preservation in calculating the disparity and diversity of early Cambrian skeletal fossils and the resulting potential effects in estimating the diversity of skeletal taxa in the Cambrian.

## Data Availability

Not applicable.
